# A multidimensional measure of polypharmacy for older adults using the Health and Retirement Study

**DOI:** 10.1038/s41598-021-86331-x

**Published:** 2021-04-22

**Authors:** Ewan Carr, Alex Federman, Olubanke Dzahini, Richard J. Dobson, Rebecca Bendayan

**Affiliations:** 1 Department of Biostatistics and Health Informatics, Institute of Psychiatry, Psychology and Neuroscience, King’s College London, London, UK; 2 Division of General Internal Medicine, Department of Medicine, Icahn School of Medicine at Mount Sinai, New York, NY USA; 3 Pharmacy Department, South London and Maudsley NHS Foundation Trust, London, UK; 4 Institute of Pharmaceutical Science, King’s College London, London, UK; 5 NIHR Biomedical Research Centre at South London and Maudsley NHS Foundation Trust and King’s College London, London, UK; 6 Institute of Health Informatics, University College London, 222 Euston Road, London, UK; 7 Health Data Research UK London, University College London, 222 Euston Road, London, UK

**Keywords:** Diseases, Health care, Medical research, Risk factors

## Abstract

Polypharmacy is commonly defined based on the number of medications taken concurrently using standard cut-offs, but several studies have highlighted the need for a multidimensional assessment. We developed a multidimensional measure of polypharmacy and compared with standard cut-offs. Data were extracted for 2141 respondents of the 2007 Prescription Drug Survey, a sub-study of the Health Retirement Study. Latent classes were identified based on multiple indicators of polypharmacy, including quantity, temporality and risk profile. A four-class model was selected based on fit statistics and clinical interpretability: ‘High risk, long-term’ (Class 1), ‘Low risk, long-term’ (Class 2), ‘High risk, short-term’ (Class 3), and ‘High risk for drug interactions, medium-term, regular’ (Class 4). Classes differed regarding sex, cohabitation, disability and multimorbidity. Participants in the ‘low risk’ class tended to be male, cohabitating, and reported fewer health conditions, compared to ‘high risk’ classes. Polypharmacy classes were compared to standard cut-offs (5+ or 9+ medications) in terms of overlap and mortality risk. The three ‘high risk’ classes overlapped with the groups concurrently taking 5+ and 9+ medications per month. However, the multidimensional measure further differentiated individuals in terms of risk profile and temporality of medication taking, thus offering a richer assessment of polypharmacy.

## Introduction

Polypharmacy is increasingly prevalent among older adults and is associated with a range of adverse health outcomes^1,2^. Polypharmacy is commonly understood as the concurrent use of multiple medications by one individual but there is no standard agreed definition and measurement varies across studies^3,4^. The most common approach is to define polypharmacy based on the number of medications taken, with a threshold of four or more^5^, five or more^6–8^, ten or more^9,10^ or other cut-points^11–13^. However, recent studies have highlighted the limitations of these ‘cut-point’ measures and emphasised the need to move towards more multidimensional assessments of polypharmacy. For example, incorporating the clinical appropriateness of prescribed medications or potential adverse drug interactions^4,14^. Thus, further research is needed to develop more comprehensive assessments of polypharmacy whilst ensuring that derived measures remain practical for clinicians and researchers.

Recent reviews^15^ have highlighted the following dimensions as key to understanding polypharmacy and its association with adverse outcomes: (1) temporality (i.e., how long the patient has been taking a particular medication); (2) dosage form (e.g., oral, topical); (3) appropriateness of the prescribed medication; (4) anticholinergic properties of the medication; and (5) potential drug interactions.

Temporality and dosage form are well known to be associated with medication adherence^15,16^. For example, Monégat et al.^3^ propose combining these indicators to define polypharmacy as continuous (e.g. number of medications prescribed more than twice a year), cumulative (e.g. average number of medications prescribed over quarters of a year) and simultaneous (e.g. total prescriptions per day).

Regarding appropriateness, several tools have been developed for assessing the appropriateness of medications for older adults including the Beers criteria^17^ and STOPP/START tool (Screening Tool of Older Person’s Prescriptions/Screening Tool to Alert doctors to Right Treatment)^18^. The Beers criteria is a widely used consensus criteria for medication use in older adults which describes potentially inappropriate drugs for this population based on their high risk of adverse outcomes such as falls and mortality^17,19,20^. The Beers criteria provide information about drug–drug and drug–disease interactions and highlight anticholinergic medications for their adverse effects linked to impaired cognitive and physical function, and risk of dementia^21,22^. The STOPP/START criteria is comparable to Beers criteria when in clinical inpatient settings but some studies suggest that adaptations are needed if these are to be applied in broader population settings^23^.

In summary, some studies support the use of cut-point measures, based on their ability to predict adverse health outcomes, such as frailty, disability, and mortality. However, others argue that limiting the assessment of polypharmacy to ‘quantity’ only is an oversimplification and omits important aspects such as appropriateness or drug interactions. The objectives of this study, therefore, are to: (1) Develop a multidimensional measurement of polypharmacy capturing temporality, dosage form and risk profile, as well as quantity; (2) Compare this multidimensional approach with standard cut-offs based on the number of medications in terms of overlap and predictive ability.

## Methods

### Sample

Data were from the 2007 Prescription Drug Survey (PDS), a sub-study of the Health Retirement Study (HRS)^24^. The HRS has been administered every 2 years since 1992 and provides representative data on adults aged 50 and over living in the United States. We supplemented information on medication use from the PDS with (i) baseline covariates from the 2006 h survey, and (ii) mortality over follow-up from the RAND-HRS Longitudinal File (2008–2016)^25,26^. Of 3536 respondents to the 2007 PDS (response rate 74%), we excluded participants missing information on current medications, those not taking any medications, and those taking > 25 medications/month. We focused our analysis on individuals aged 50–80 years, since individuals older than 80 constitute a different population, the oldest-old, with differentiated patterns of polypharmacy and higher risk of adverse outcomes related to polypharmacy^27^ The analytical sample therefore contained 2141 individuals. For Cox proportional hazard models, described below, we further excluded participants missing information on mortality over follow-up reducing the same size for these analyses to 1935.

### Measures

#### Polypharmacy indicators

We identified eight indicators split over three domains based on the previous literature review^4,17^ and discussion with expert clinicians and pharmacists. ‘Temporality’ included two indicators: (1) Percentage of prescription medications taken on a regular basis (every day or every week) rather than taken as needed. This categorisation was based on participant self-report in the PDS questionnaire where participants were asked “Of those prescription drugs, how many are ones you take on a regular basis (for example, every day or every week)?” Since most medications were taken regularly, this variable was dichotomised as ‘Up to 75%’ vs. ‘More than 75%’. (2) Duration of medication use, assessed based on participant self-report in the PDS questionnaire where participants were asked how long they had been taking each prescription medication. We summarised this information by counting the number of medications they had been taking for 0–5 months, 6–24 months, and 25 + months. ‘Risk profile’ included three indicators: (3) Number of anticholinergic medications currently taken; (4) number of potentially inappropriate medications currently taken; and (5) number of potential adverse drug interactions. For these measures, we selected the 90% most common medications (219 medications, out of 608 in total) and categorised each medication based on published studies (Tables 7, 2, and 5 from the 2019 Beers criteria^17^, respectively) and manual coding by an expert pharmacist. Due to small cell counts, all three measures were dichotomised as 0 vs. 1 + medications. Finally, ‘Quantity’ included three indicators derived from participant self-report in the PDS questionnaire: Number of (6) prescription and (7) non-prescription medications used in the last month; and (8) total number of different dosage forms used for current medications (e.g. tablet, liquid, inhalant, other).

#### Covariates for use in prediction modelling

Age was derived from birth date as the participant’s age on October 1st 2007 (the month when PDS questionnaires were sent out). Sex, ethnicity, education, cohabitation (living with partner vs. not living with partner), chronic health conditions, and Activities of Daily Living (ADLs) were derived from the 2006 h interview. Ethnicity was coded into three categories (White/Caucasian, Black/African American and Other). Education was coded into five categories (less than high-school, GED, high school, some college, above college). GED refers to ‘General Educational Development’, an alternative to the high school diploma. Chronic health conditions were measured as the total number of conditions as well as binary indicators of specific conditions: cancer (malignant tumor excluding skin), lung disease, diabetes, joint disease (arthritis, connective tissue disease), heart problems (myocardial infarction, angina, congestive heart failure, arrhythmia, other heart problems), stroke, high blood pressure, or depression caseness. Depression caseness was quantified as a score of four or greater on the Center for Epidemiologic Studies Depression Scale (CES-D). In addition, we derived a three-category variable to capture multimorbidity (‘no multimorbidity’ = 0–1 conditions; ‘simple multimorbidity’ = 2–3 conditions; and ‘complex multimorbidity’ = 4 + conditions). Previous research has shown that differentiating between simple and complex multimorbidity can better capture the variability in multimorbidity in older adults^28,29^. ADLs^30^ included walking across the room, dressing, bathing and showering, eating, and getting in and out of bed. To ease interpretation and for consistency with previous research in older adults^31^ this variable was dichotomised as 0 (‘No difficulties’) or 1 (‘Reported difficulties in at least one of the tasks’).

#### Mortality

Date of death was derived from the RAND-HRS Longitudinal File, which incorporates successive biennial HRS interviews between 2008 and 2016. Year and month of death were ascertained from the participant’s exit interview or spouse.

### Statistical analyses

The analysis was in two parts. First, to derive a multidimensional measure of polypharmacy we used latent class analysis (LCA) to identify subgroups of participants based on their response patterns across eight polypharmacy indicators. We considered models with 1–10 latent classes, estimated using Mplus 8.2^32^. The optimal number of classes was chosen based on clinical interpretability and model fit [Akaike information criterion (AIC) and sample size adjusted Bayesian information criterion (BIC)]. Second, we used Cox proportional hazards models to assess whether polypharmacy was predictive of mortality over follow-up. We compared polypharmacy defined using cut-points based on the number of prescription medications taken per month, either (i) 5 + vs. fewer or (ii) 9 + vs. fewer; or (iii) membership to polypharmacy latent classes for the chosen LCA model. Class membership was measured using binary indicators (0 vs. 1) for each latent class. We chose the 5 + cut-point because this is most commonly used in past studies^15^; we additionally considered the 9 + cut-point to reflect growing rates of polypharmacy in recent years. Predictive performance was assessed based on the C-index with adjustment for right-censoring^33^ using tenfold nested cross-validation with 100 repeats. Cox proportional hazards models were adjusted for age, sex, education, ethnicity, and chronic health conditions, as detailed above. Missing covariate information was imputed using multivariate imputation^34^. Prediction models were estimated using Scikit-learn^35^ and Scikit-Survival^36^ in Python 3.8^37^.

### Sensitivity analyses

We conducted two sensitivity analyses to consider whether our findings were influenced by the chosen age criteria (ages 50–80). Firstly, since the Beers criteria were originally intended for use in adults aged 65 and older, we repeated the latent class analysis after excluding younger participants (aged 50–64). Secondly, to consider whether including older participants (aged over 80) would produce different classes, we repeated the analyses for all participants aged 50 and over.

## Results

### Descriptive summary of analytical sample

The analytical sample contained 2141 respondents, having removed those missing information on current medications (n = 536), younger than 50 years (n = 10) or older than 80 years (n = 815), not taking any medications (n = 30), or taking > 25 medications/month (n = 4). Compared to excluded participants, the analysed sample tended to be younger (mean 74.6 vs. 71.0; p < 0.001) but were similar in terms of sex and ethnicity (see Supplementary Table 2 for details). The sample contained slightly more women than men (n = 1244; 58%) and was predominantly White/Caucasian (n = 1759; 82%). Most participants reported at least one chronic condition (n = 1961; 92%) and 17% reported difficulties with ADLs (n = 367).

### Polypharmacy classes

A four-class model was selected based on model interpretability and model fit (AIC and adjusted BIC; see Supplementary Table 1). Below we describe the classes labelled by their distinguishing polypharmacy characteristics (see Table 1 for details):

**Table 1:** Means and percentages of latent class indicators for 4-class model.

Latent class indicator	Class 1	Class 2	Class 3	Class 4	p-value for differences across classes^a^
	(n = 484)	(n = 1331)	(n = 126)	(n = 200)	
**Means**					
Number of non-prescription drugs taken this month	1.3	1.2	1.5	1.3	0.017
Number of prescription drugs taken this month	8.1	3.3	6.9	7.7	< 0.001
Number of routes of administration	2.3	1.5	2.2	2.3	< 0.001
**Of current prescription drugs, number taken for**					
0–5 months	0.3	0.3	3.3	0.5	< 0.001
6–24 months	1.2	0.8	1.2	5.0	< 0.001
25 + months	5.9	1.8	1.7	1.8	< 0.001
**Percentages**					
Taking 1 + anticholinergic medication	19%	4%	20%	18%	< 0.001
Taking 1 + inappropriate medication	44%	17%	43%	37%	< 0.001
1 + potential drug interactions, among current prescription medications	20%	1%	16%	32%	< 0.001
Taking > 75% of current prescription medications regularly	0.90	0.87	0.84	0.91	0.001

^a^p-values based on one-way analysis of variance, estimated separately for each latent class indicator.

#### Class 1 ‘High risk, long-term’ (n = 484)

Individuals in this class tended to have been taking medications for a longer duration (> 2 years), and took a higher number of prescribed medications (8.1 per month, compared to 3.3, 6.9 and 7.7 for other classes). Regarding risk profile, this class was more likely to be taking inappropriate medications than other classes (44% taking at least one, compared to 17–43% in other classes) and had high risk associated with anticholinergic (19%) and drug interactions (20%).

#### Class 2 ‘Low risk, long-term’ (n = 1331)

Individuals in this class took fewest prescription medications (3.3 per month, compared with 6.9 or more in other classes) and had the lowest risk profile in terms of anticholinergic, inappropriate, drug interactions. Just 4% were taking one or more anticholinergic medications (compared to 18% or more in other classes) and 17% were taking inappropriate medications (compared to 37% or more in other classes). Most medications were taken for long durations (> 25 months).

#### Class 3 ‘High risk, short-term’ (n = 126)

This class took a high number of both prescription and non-prescription medications each month (6.9 and 1.5, respectively), but tended to take medications on a short-term basis (< 6 months). They had a high risk profile regarding anticholinergic (20% taking at least one) and inappropriate (43% taking at least one) medications and a moderate risk regarding drug interactions.

#### Class 4 ‘High risk for drug interactions, medium-term, regular’ (n = 120)

Individuals in this class took a high number of prescription medications (7.7 per month), most of which they had been taking regularly for between 6 months and 2 years. They were most likely to have one or more drug interactions (32% compared to 1–20% in other classes) and had a moderate-to-high risk profile regarding anticholinergic and inappropriate medications (18% and 37% taking at least one, respectively).

These classes were similar in terms of age and ethnicity (Table 2). Those in Class 2 (‘Low risk, long-term’) were more likely to be male (44% vs. 39% or less in other classes; p = 0.027) and cohabiting (69% vs. 64% or less in other classes; p = 0.012). There were also differences regarding disability, with those in Class 2 reporting fewer chronic conditions and being less likely to report difficulties with ADLs (11% vs. 22% or more; p < 0.001), compared to other classes. In contrast, those in Class 4 (‘High risk for drug interactions, medium-term, regular’) were most likely to report difficulties with ADLs (35%; p < 0.001).

**Table 2:** Demographic and clinical characteristics of analytical sample stratified by polypharmacy subgroups (n = 2141).

	Overall	Characteristics by polypharmacy status										
		Number of concurrent prescription medications						Latent classes (4-class solution)				
		5 + medications			9 + medications							
		< 5	5 +	p-value (DF)	< 9	9 +	p-value (DF)	Class 1	Class 2	Class 3	Class 4	p-value (DF)
	n = 2141	n = 1142	n = 999		n = 1857	n = 284		n = 484	n = 1331	n = 126	n = 200	
Age, mean (SD)	71.0 (5.1)	71.0 (4.8)	71.1 (5.5)	p = 0.518 (1)	71.1 (5.0)	70.5 (5.8)	p = 0.049 (1)	71.0 (5.6)	71.1 (4.8)	70.8 (5.6)	70.7 (5.7)	p = 0.652 (3)
Female gender, N (%)	1244 (58)	641 (56)	603 (60)	p = 0.053 (1)	1061 (57)	183 (64)	p = 0.023 (1)	296 (61)	741 (56)	78 (62)	129 (64)	p < 0.001 (3)
**Ethnicity N (%)**												
White/Caucasian	1759 (82)	961 (84)	798 (80)	p = 0.011 (2)	1532 (82)	227 (80)	p = 0.033 (1)	394 (81)	1112 (84)	97 (77)	156 (78)	p = 0.167 (6)
Black/African American	298 (14)	135 (12)	163 (16)		247 (13)	51 (18)		72 (15)	171 (13)	22 (17)	33 (16)	
Other	84 (4)	46 (4)	38 (4)		78 (4)	6 (2)		18 (4)	48 (4)	7 (6)	11 (6)	
Cohabiting, N (%)	1405 (66)	771 (69)	634 (64)	p = 0.028 (1)	1232 (67)	173 (61)	p = 0.061 (1)	311 (65)	902 (69)	75 (60)	117 (59)	p = 0.002 (3)
**Number chronic conditions, N (%)**												
0–1	598 (28)	476 (42)	122 (12)	p < 0.001 (2)	583 (31)	15 (5)	p < 0.001 (2)	43 (9)	500 (38)	26 (21)	29 (14)	p < 0.001 (6)
2–3	1138 (53)	579 (51)	559 (56)		1007 (54)	131 (46)		264 (55)	701 (53)	70 (56)	103 (52)	
3 +	405 (19)	87 (8)	318 (32)		267 (14)	138 (49)		177 (37)	130 (10)	30 (24)	68 (34)	
**Specific chronic conditions, N (%)**												
High blood pressure	1407 (67)	628 (56)	779 (79)	p < 0.001 (1)	1168 (64)	239 (85)	p < 0.001 (1)	379 (79)	790 (60)	84 (68)	154 (78)	p < 0.001 (3)
Diabetes	497 (24)	136 (12)	361 (36)	p < 0.001 (1)	353 (19)	144 (51)	p < 0.001 (1)	200 (41)	202 (15)	38 (31)	57 (29)	p < 0.001 (3)
Cancer	347 (16)	180 (16)	167 (17)	p = 0.642 (1)	290 (16)	57 (20)	p = 0.077 (1)	88 (18)	207 (16)	18 (15)	34 (17)	p = 0.304 (3)
Lung disease	246 (12)	70 (6)	176 (18)	p < 0.001 (1)	177 (10)	69 (25)	p < 0.001 (1)	90 (19)	91 (7)	22 (18)	43 (22)	p < 0.001 (3)
Heart problem	569 (27)	175 (16)	394 (40)	p < 0.001 (1)	418 (23)	151 (54)	p < 0.001 (1)	216 (45)	252 (19)	35 (28)	66 (34)	p < 0.001 (3)
Stroke	190 (9)	64 (6)	126 (13)	p < 0.001 (1)	140 (8)	50 (18)	p < 0.001 (1)	75 (16)	80 (6)	12 (10)	23 (12)	p < 0.001 (3)
Arthritis	1407 (67)	662 (59)	745 (75)	p < 0.001 (1)	1181 (65)	226 (80)	p < 0.001 (1)	368 (76)	785 (60)	94 (76)	160 (81)	p < 0.001 (3)
CES-D depression, N (%)	327 (16)	134 (12)	193 (20)	p < 0.001 (1)	p < 0.001 (1)	67 (25)	p < 0.001 (1)	90 (19)	159 (13)	26 (22)	52 (28)	p < 0.001 (3)
Difficulties with 1 + ADLs, N (%)	367 (17)	122 (11)	245 (25)	p < 0.001 (1)	267 (15)	100 (35)	p < 0.001 (1)	128 (27)	143 (11)	27 (22)	69 (35)	p < 0.001 (3)

With regards to health conditions, Class 1 (‘High risk, long-term’) saw the highest levels of overall multimorbidity, with 37% reporting 4 or more conditions (compared to 10–34% in other classes; p < 0.001) and just 9% reporting 0 or 1 conditions (compared to 14–38%; p < 0.001). When we examined specific health conditions independently, we found that individuals in Class 2 (‘Low risk, long-term’) reported the lowest rates for almost all conditions. For example, 15% in Class 2 reported diabetes, compared to 29–41% in other classes (p < 0.001); and 13% reported CES-D depression, compared to 19–28% in other classes (p < 0.001). Among the other classes, rates of almost all specific conditions were highest in Class 4 (‘High risk for drug interactions, medium-term, regular’), whereas Class 3 (‘High risk, short-term’) showed lower percentages for high blood pressure (68%), heart problems (28%) and stroke (10%) when compared with Class 1 and Class 4. Post-hoc pairwise comparisons showed that differences between pairs of classes were statistically significant.

There was considerable overlap between the four polypharmacy classes and the cut-point measure based on 5 + or 9 + concurrent medications. Of those in Class 2 (‘Low risk, long-term’), only 20% were taking 5 + medications per month, compared to 96%, 75%, and 90% of participants in the three ‘high risk’ classes (Classes 1, 3, and 4, respectively); and just 1% were taking 9 + medication (compared to 35%, 28% and 34% in Classes 1, 3 and 4, respectively). Therefore, the four-class polypharmacy measure differentiates not only between low and high risk polypharmacy (Class 2 vs. Classes 1, 3 or 4) but further distinguishes different types of high risk polypharmacy, as we discuss below.

### Predicting mortality over follow-up

Our multidimensional measure of polypharmacy and the 5 + and 9 + cut-off measures were similar in their ability to predict mortality over follow-up (2007–2016). The C-index scores for latent class, 5 + and 9 + measures were 0.676, 0.678, and 0.674, respectively, after adjusting for age, sex, education, ethnicity, and chronic health conditions. Notably, polypharmacy was one of the strongest predictors of mortality risk over follow-up, regardless of the measure used. Membership in Class 1 (‘High risk, long-term’) and Class 4 (‘High risk for drug interactions, medium-term, regular’) was strongly associated with increased risk of mortality, compared to Class 2 [Hazard Ratio (HR) for Class 1 = 1.52; 95% CI 1.23, 1.88; HR for Class 4 = 1.85; 95% CI 1.42, 2.40]. Similarly, participants taking 5 + (HR 1.57; 95% CI 1.29, 1.90) or 9 + medications (HR 1.59; 95% CI 1.27, 1.98) experienced substantially higher mortality risks compared to those taking fewer (see Supplementary Table 3 for details).

### Sensitivity analyses

We found consistent results when repeating the latent class analysis after (i) excluding younger participants (ages 65 +; Supplementary Table 4) or (ii) including older participants (ages 50 +; Supplementary Table 5). The number of classes, clinical interpretability, and model fit were almost unchanged and the composition of classes in terms of polypharmacy indicators and clinical characteristics was very similar.

## Discussion

This study aimed to develop a multidimensional measure of polypharmacy and compare this with a standard cut-point based on taking five or more medications. A four-class model was chosen based on model fit and clinical interpretability, which overlapped with the standard cut-point measure. Our measure distinguished polypharmacy subtypes that differed with regards to quantity, temporality and risk profile. The largest class (Class 2, ‘Low risk, long-term’) tended to be healthier, took fewer medications and had a lower risk profile, compared to other classes. Most individuals in this class were below standard cut-offs for polypharmacy (< 5 medications). By contrast, most individuals in the other three classes all exceeded standard thresholds for polypharmacy. These three classes differ in terms of the potential drivers of this risk and how long have they been taking these medications. For example, Class 1 (‘High risk, long-term’) had the highest percentage of risk associated with anticholinergic and inappropriate medications, while Class 4 (‘High risk for drug interactions, medium-term, regular’) had the highest percentage of risk associated with drug interactions. These findings are in line with Wastesson et al.^4^ who highlighted the need for more holistic assessments of polypharmacy, including other relevant indicators such as adverse interactions.

Besides risk profile, the other dimension that distinguished classes was duration. Among the three ‘high risk’ classes, individuals in Class 3 (‘High risk, short-term’) tended to take medications for much shorter durations, compared to other classes. The role of duration as a possible differentiating feature links with adherence^16^ and the on-going debate around deprescribing (the gradual withdrawal from medications with healthcare supervision) which is key to personalized medicine. While research interest in deprescribing has recently grown^38^, it remains limited in practice by social factors such as specialist or nurse influences^39^, and more research should be performed to examine whether duration is key for deprescribing guidelines for some specific drugs or not. It is important to note that duration of treatment is not a sufficient criterion for deprescribing and only is suitable when it targets drugs that are potentially inappropriate, for which the risks outweigh the benefits. Therefore, individuals taking medications for longer periods of time could be targeted for deprescribing drugs that are potentially inappropriate. Our measure could be used to identify these individuals, for example, prioritizing individuals classified in Class 1 ‘High risk, long-term’.

Across polypharmacy classes, individuals were similar in age and ethnicity but differed in terms of sex, cohabitation, disability and health conditions. Those in Class 2 (‘Low risk, long-term’) were more likely to be male and cohabiting, report fewer chronic conditions and difficulties with activities of daily living. These findings are consistent with previous studies showing no differences in polypharmacy by ethnicity^40^ and higher rates of polypharmacy among women^41,42^ or separated or never married individuals^43^. In addition, we found significant differences in disability and health conditions among the ‘high risk’ classes. For disability, Class 4 (‘High risk for drug interactions, medium-term, regular’) was most likely to report difficulties with ADLs, compared to other classes. While for multimorbidity, although all ‘high risk’ classes were more likely to have two or more co-existing health conditions, individuals in Class 1 (‘High risk, long-term’) were more likely to have complex multimorbidity (4 or more conditions). These results are in line with previous research that has found that individuals with polypharmacy are more likely to have difficulties with ADLs and more likely to have two or more chronic health conditions^44,45^. Moreover, the link between high risk polypharmacy (Class 1) and complex multimorbidity (4 or more conditions) is of particular clinical relevance, since these individuals are more likely to have higher healthcare needs and may be using multiple healthcare providers. It should be noted that most previous research uses polypharmacy standard cut-offs, making it difficult to draw direct comparisons. Further research exploring differences between the ‘high risk’ classes should be performed in other populations. For example, recent research showing sex differences in polypharmacy are specific to older adults aged 50–80, but this pattern seems to reverse among those over 80, the oldest old^45^.

With regards to the comparison of the multidimensional and the cut-point measures of polypharmacy, the three ‘high risk’ classes (Classes 1, 3, and 4) overlapped substantially with the groups taking 5 + and 9 + medications per month, whereas most members of Class 2 (‘Low risk, long term’) were taking fewer than five medications per month. When we examined the predictive value of the proposed multidimensional measure, we found that it predicted mortality even after adjusting for demographic characteristics and co-existing health conditions. This suggests that although multimorbidity is known to be inherently associated with polypharmacy^46^, polypharmacy has an independent role in its association with mortality. However, it is notable that our multidimensional measure and cut-off measure (5 + and 9 +) were similar in terms of their ability to predict mortality over follow-up. This suggests that both our theoretically derived multidimensional measure or standard cut-offs can be used for mortality risk stratification purposes. As our results highlight, researchers without detailed information on polypharmacy may apply standard cut-offs, but these definitions may overlook differences in the risk profile of these individuals for other health outcomes, such as hospital admission or need for long-term care.

This is the first study to our knowledge to provide evidence of a multidimensional measure of polypharmacy using data from a large population study and its main strengths can be summarized in the following three points. Firstly, these findings further our understanding of the polypharmacy construct in older adults by providing empirical evidence to the on-going debate around the need for multidimensional approaches that go beyond quantity. By using a large population study and dimensions that can be extracted from other population data sources and electronic health records, our measure can be replicated and compared in other settings. We expect that this study will facilitate cross-cohort comparisons and further work in this direction. Finally, this measure has the potential to be used in translational medicine in the development of new screening tools to identify individuals in need of clinical reviews of their medication regimen. For example, it could be embedded in pipelines in hospital settings to support clinical decision making. However, further research will have to follow up to ensure its direct clinical applicability.

However, several limitations must also be acknowledged. First, data were self-reported which may introduce potential biases, such as recall or social desirability. Future studies should replicate our findings using objective measures, for example, extracted from electronic health records. Secondly, our data were drawn from a population aged 50 years and over and while polypharmacy is common in older adults who are more likely to have two or more chronic health conditions (i.e. multimorbidity), research has highlighted socially deprived individuals are likely to develop multimorbidity earlier in life^47^ and could also be taking multiple medications. Thus, further studies with younger populations or even specific sub-populations traditionally neglected in large population studies (e.g. those with mental health disorders) should be considered. While the Beers criteria were originally intended for populations over 65 years old, results from our sensitivity analyses suggest they may be useful for younger populations, as some past studies have suggested^48^. Thirdly, our data were drawn from the United States and these analyses should be replicated in other countries to allow comparison of prescription guidelines. For example, the Beers criteria are not often used outside the USA, in part due to differences between national drug formularies (i.e. differences in approved and utilized drugs) between countries. Therefore, future studies should therefore investigate the potential transferability of Beers criteria for cohorts over 65 or outside the USA.

Fourth, although we focused on indicators likely to be available in routine clinical datasets to ensure replicability and potential for implementation, we did not include other complex measures of medication regimen such as the medication regimen complexity index or the drug burden index or considered the potential benign impact of some medication^12^. Other measures to address alternative dimensions of polypharmacy include the medication regimen complexity index (MRCI)^49^, the drug burden index (DBI)^50^, and the medication appropriateness index (MAI)^51,52^. However, these cannot be directly compared to our measure as some are broader (MRCI) and others are more specific (DBI and MAI). The MRCI is a 65-item instrument with different complexity levels which are based on weighted averages of the number of drugs, dosage frequency, administration instructions, and prescribed dosage forms. Although previous research shows that it is a good predictor of hospitalization, hospital readmission, and medication adherence^53^, recent reports have highlighted that the MRCI is a long and complex instrument that does not outperform patient medication counts to predict medication-related hospital admission^54^. The DBI, which also has been found to be associated with functioning outcomes in older adults^55^, is a more specific measure that captures overall exposure to medications with anticholinergic and sedative properties that implements the principle of dose response to determine the effect of medication exposure. However, this measure does not account for other relevant factors for research in older adults (e.g., multimorbidity^56^). The MAI has shown to be also a valuable research tool for measuring potentially inappropriate prescribing in the elderly, and especially sensitive for those with higher levels of inappropriateness. However, it does not address some aspects of suboptimal prescribing (i.e., polypharmacy or underuse of medically necessary medications) and its evaluation requires an expert clinician, can be time consuming, and it may be subject to reliability issues when more than one evaluator is used^57^. Future studies should explore the potential convergent or divergent validity of these scales against our proposed measure and compare its applicability with clinical data sources.

Fifth, polypharmacy is variable over time^58,59^ and several concepts of polypharmacy require longitudinal data to explore transitions between drugs, the duration of use, total quantity of drugs used or pauses in treatment. Although we considered treatment duration estimated by the participants, data remain limited to drugs that were used during the study period, and the quality of adherence to therapy cannot be assessed. Future longitudinal studies should be performed to address some of these questions. Finally, we used the most recent Beers criteria for assessing appropriateness and drug interactions^17^, however these criterion change over time so future research will need to include updates.

To sum up, our multidimensional measure provided a more nuanced characterisation of polypharmacy that allowed us to group individuals in a richer and more meaningful way than when using standard cut-offs. The multidimensional measure and standard cut-offs were similar in terms of mortality risk stratification, but for studies addressing outcomes besides mortality, we believe that the multidimensional measure can provide novel insights. Despite differences in data collection between sites or countries, many of the included indicators could be extracted from comparable population datasets, or potentially, from electronic health records supported by future algorithm developments such as natural language processing. While further research is needed to understand the clinical applicability of a multidimensional measure of polypharmacy, it may also be possible to develop novel artificial intelligence pipelines to detect individuals in need of clinical review of their medication regimen.

## Supplementary Information

Supplementary Information.

Supplementary Table 1.

Supplementary Table 2.

Supplementary Table 3.

Supplementary Table 4.

Supplementary Table 5.

## Data Availability

Data was extracted from the Health Retirement Study which is a publicly available dataset. More details in https://hrs.isr.umich.edu/data-products/access-to-public-data.
