# Supplementary material for: A multidimensional measure of polypharmacy for older adults using the Health and Retirement Study
Source: Sci Rep. 2021 Apr 22;11:8783. doi: 10.1038/s41598-021-86331-x (PMC8062687; doi:10.1038/s41598-021-86331-x)
Supplement: Supplementary file 1 — Supplementary Information. [file 41598_2021_86331_MOESM1_ESM.docx]

**TITLE: A multidimensional measure of polypharmacy for older adults using the Health and Retirement Study**

RUNNING HEAD: A multidimensional measure of polypharmacy

**Authors:** Carr, E.^1,5^, Federman, A^2^., Dzahini, O.^3, 4^, Dobson, R.J.^1,5,6,7^., Bendayan, R^1,5^.

^1^ Department of Biostatistics and Health Informatics, Institute of Psychiatry, Psychology and Neuroscience, King’s College London, London, United Kingdom

^2^ Division of General Internal Medicine, Department of Medicine, Icahn School of Medicine at Mount Sinai, New York, NY, USA

^3^ Pharmacy Department, South London and Maudsley NHS Foundation Trust, United Kingdom

^4^ Institute of Pharmaceutical Science, King’s College London, London, United Kingdom

^5^ NIHR Biomedical Research Centre at South London and Maudsley NHS Foundation Trust and King’s College London, London, United Kingdom

^6^ Institute of Health Informatics, University College London, 222 Euston Road, London, United Kingdom

^7^ Health Data Research UK London, University College London, 222 Euston Road, London, United Kingdom.

**ORCIDs:**

E. Carr 0000-0002-1146-4922

A. Federman 0000-0002-4824-064X

D. Olubanke 0000-0003-3878-2143

R. Dobson 0000-0003-4224-9245

**Corresponding author**: Rebecca Bendayan / ORCID: 0000-0003-1461-556X

E-mail: [rebecca.bendayan@kcl.ac.uk](mailto:rebecca.bendayan@kcl.ac.uk)

NIHR Maudsley Biomedical Research Centre

Department of Biostatistics & Health Informatics

SGDP Centre, IoPPN, Box PO 80

De Crespigny Park, Denmark Hill

London SE5 8AF, UNITED KINGDOM

**Supplementary materials**

Top 90% most common medications (n=219) from the 2007 Prescription Drugs Survey. These were used when deriving the number of anticholinergic medications, potentially inappropriate medications, and potential drug interactions.

Levothyroxine

Metoprolol

Lisinopril

Atorvastatin

Simvastatin

Hydrochlorothiazide

Furosemide

Atenolol

Amlodipine

Metformin

Warfarin

Potassium Chloride

Clopidogrel

Omeprazole

Lovastatin

Aspirin

Hydrochlorothiazide-Triamterene

Alendronate

Diltiazem

Valsartan

Esomeprazole

Ezetimibe-Simvastatin

Glipizide

Ezetimibe

Digoxin

Rosuvastatin

Tamsulosin

Allopurinol

Albuterol

Gabapentin

Carvedilol

Losartan

Fluticasone-Salmeterol

Risedronate

Acetaminophen-Hydrocodone

Glyburide

Pioglitazone

Pravastatin

Enalapril

Ranitidine

Verapamil

Nifedipine

Celecoxib

Terazosin

Pantoprazole

Sertraline

Hydrochlorothiazide-Lisinopril

Lansoprazole

Alprazolam

Amlodipine-Benazepril

Ramipril

Conjugated Estrogens

Glimepiride

Clonidine

Hydrochlorothiazide-Valsartan

Escitalopram

Montelukast

Acetaminophen-Propoxyphene

Fenofibrate

Prednisone

Isosorbide Mononitrate

Latanoprost Ophthalmic

Lorazepam

Nitroglycerin

Doxazosin

Fexofenadine

Tramadol

Naproxen

Donepezil

Irbesartan

Spironolactone

Tolterodine

Zolpidem

Folic Acid

Tiotropium

Meloxicam

Quinapril

Cetirizine

Felodipine

Olmesartan

Amitriptyline

Fluticasone Nasal

Paroxetine

Albuterol-Ipratropium

Fluoxetine

Hydrochlorothiazide-Losartan

Ibuprofen

Citalopram

Multivitamin

Cyclobenzaprine

Estradiol

Pregabalin

Clonazepam

Insulin Glargine

Niacin

Propranolol

Glyburide-Metformin

Meclizine

Trazodone

Rosiglitazone

Benazepril

Finasteride

Gemfibrozil

Raloxifene

Dutasteride

Ibandronate

Oxybutynin

Ferrous Sulfate

Rabeprazole

Venlafaxine

Diclofenac

Memantine

Travoprost Ophthalmic

Carbidopa-Levodopa

Timolol Ophthalmic

Fosinopril

Docusate

Amiodarone

Bimatoprost Ophthalmic

Bupropion

Mometasone Nasal

Atenolol-Chlorthalidone

Diazepam

Temazepam

Loratadine

Colchicine

Duloxetine

Alfuzosin

Amlodipine-Atorvastatin

Amoxicillin

Fluvastatin

Insulin Isophane

Methotrexate

Acetaminophen

Bisoprolol-Hydrochlorothiazide

Brimonidine Ophthalmic

Candesartan

Dorzolamide-Timolol Ophthalmic

Etodolac

Nitrofurantoin

Phenytoin

Hydrochlorothiazide-Irbesartan

Hydrochlorothiazide-Olmesartan

Multivitamin With Minerals

Quetiapine

Sitagliptin

Telmisartan

Acetaminophen-Oxycodone

Alendronate-Cholecalciferol

Calcium Carbonate

Hydralazine

Hydroxychloroquine

Polyethylene Glycol 3350

Insulin Lispro

Labetalol

Metoclopramide

Sotalol

Anastrozole

Aspirin-Dipyridamole

Bumetanide

Calcitonin

Indapamide

Mirtazapine

Nisoldipine

Nortriptyline

Cilostazol

Ciprofloxacin

Eszopiclone

Famotidine

Ipratropium

Nadolol

Omega-3 Polyunsaturated Fatty Acids

Oxycodone

Desloratadine

Hydroxyzine

Insulin Isophane-Insulin Regular

Mometasone

Nabumetone

Thyroid Desiccated

Captopril

Dicyclomine

Insulin Aspart

Azelastine Nasal

Cyanocobalamin

Divalproex Sodium

Metolazone

Solifenacin

Torsemide

Baclofen

Chlorthalidone

Cimetidine

Conjugated Estrogens-Medroxyprogesterone

Fluticasone

Pentoxifylline

Pramipexole

Rivastigmine

Ropinirole

Acetaminophen-Tramadol

Benazepril-Hydrochlorothiazide

Carisoprodol

Darifenacin

Diclofenac-Misoprostol

Exenatide

Formoterol

Hyoscyamine

Indomethacin

Lamotrigine

Letrozole

Lovastatin-Niacin

Metronidazole Topical

Propafenone

Propoxyphene

Risperidone

Topiramate

Budesonide

Calcium-Vitamin D

Cephalexin

Doxepin

Hydrochlorothiazide-Spironolactone
