# Supplementary material for: A multidimensional measure of polypharmacy for older adults using the Health and Retirement Study
Source: Sci Rep. 2021 Apr 22;11:8783. doi: 10.1038/s41598-021-86331-x (PMC8062687; doi:10.1038/s41598-021-86331-x)
Supplement: Supplementary file 5 — Supplementary Table 4. [file 41598_2021_86331_MOESM5_ESM.docx]

**Supplementary Table 4.** Composition of latent classes for sensitivity analysis restricted to participants aged 65+ (n=2738)

|  |  | Class 1 | Class 2 | Class 3 | Class 4 |
| --- | --- | --- | --- | --- | --- |
| Latent class indicator | | (n=630) | (n=1679) | (n=172) | (n=257) |
|  |  | *Means* | | | |
| Number of non-prescription drugs taken this month | | 1.3 | 1.2 | 1.4 | 1.4 |
| Number of prescription drugs taken this month | | 7.9 | 3.2 | 7.3 | 7.1 |
| Number of routes of administration | | 2.3 | 1.5 | 2.2 | 2.3 |
| Of current prescription drugs, number taken for: | |  |  |  |  |
|  | 0-5 months | 0.2 | 0.2 | 3.1 | 0.3 |
|  | 6-24 months | 1.1 | 0.8 | 1.6 | 4.9 |
|  | 25+ months | 5.9 | 1.8 | 1.9 | 1.5 |
|  |  | *Percentages* | | | |
| Taking 1+ anticholinergic medication | | 19% | 4% | 21% | 11% |
| Taking 1+ inappropriate medication | | 40% | 17% | 40% | 40% |
| 1+ potential drug interactions, among current prescription medications | | 18% | 1% | 23% | 20% |
| Taking >75% of current prescription medications regularly | | 92% | 88% | 84% | 93% |
